# Vaginal Natural Orifice Transluminal Endoscopic Surgery (vNOTES) in Risk-Reducing Gynecologic Cancer Surgery: A New Frontier in Hereditary Cancer Prevention

**DOI:** 10.3390/jcm14124018

**Published:** 2025-06-06

**Authors:** Victor Bogdan Buciu, Denis Mihai Șerban, Dorin Novacescu, Larisa Tomescu, Sebastian Ciurescu, Nicoleta Nicolae, Adrian Ratiu, Elena Lavinia Rusu, Sebastian Olariu, Mihai Ionac, Ioan Sas

**Affiliations:** 1 Doctoral School, “Victor Babes” University of Medicine and Pharmacy Timisoara, E. Murgu Square, No. 2, 300041 Timisoara, Romania; victor.buciu@umft.ro (V.B.B.); sebastian.ciurescu@umft.ro (S.C.); elena.rusu@umft.ro (E.L.R.); raul.olariu@umft.ro (S.O.); 2 Department of Obstetrics-Gynecology, Discipline of Obstetrics-Gynecology, “Victor Babes” University of Medicine and Pharmacy Timisoara, E. Murgu Square, No. 2, 300041 Timisoara, Romania; tomescu.larisa@umft.ro (L.T.); nicolae.nicoleta@umft.ro (N.N.); ratiu.adrian@umft.ro (A.R.); sas.ioan@umft.ro (I.S.); 3 Department II of Microscopic Morphology, “Victor Babes” University of Medicine and Pharmacy Timisoara, E. Murgu Square, No. 2, 300041 Timisoara, Romania; novacescu.dorin@umft.ro; 4 Department of Microsurgery, Vascular Surgery and Scientific Research Methodology, “Victor Babes” University of Medicine and Pharmacy Timisoara, E. Murgu Square, No. 2, 300041 Timisoara, Romania; mihai.ionac@gmail.com

**Keywords:** vNOTES, hereditary cancer syndromes, BRCA, Lynch syndrome, prophylactic surgery, salpingo-oophorectomy, hysterectomy, minimally invasive surgery, SEE-FIM protocol

## Abstract

**Background:** Hereditary cancer syndromes such as BRCA1/2 and Lynch syndrome significantly increase the lifetime risk of ovarian, fallopian tube, and endometrial cancers. Risk-reducing salpingo-oophorectomy (RRSO) and hysterectomy are standard preventive strategies. Vaginal natural orifice transluminal endoscopic surgery (vNOTES) has recently emerged as a minimally invasive, scarless alternative that may enhance patient acceptance while maintaining oncologic safety. **Objective:** This narrative review aims to synthesize the current evidence regarding the role of vNOTES in risk-reducing gynecologic surgery for women with hereditary cancer syndromes, focusing on surgical feasibility, technical considerations, oncologic safety, and patient-reported outcomes. **Methods:** A structured literature search was conducted in PubMed and Web of Science for studies published between January 2000 and April 2025, using terms related to vNOTES, prophylactic gynecologic surgery, BRCA mutations, and Lynch syndrome. Inclusion criteria focused on studies reporting outcomes of vNOTES in risk-reducing or oncologic contexts. A total of eight studies were included for qualitative synthesis. **Results:** vNOTES has demonstrated technical feasibility and favorable surgical outcomes in risk-reducing procedures such as RRSO and hysterectomy in BRCA and Lynch syndrome carriers. Comparative studies report lower postoperative pain, faster recovery, and high patient satisfaction, with oncologic standards maintained through specimen containment, peritoneal inspection, and adherence to the SEE-FIM protocol. Limitations include the learning curve and restricted access to the upper abdomen, which may necessitate hybrid approaches in selected cases. **Conclusions:** vNOTES offers a promising, patient-centered surgical approach for hereditary cancer prevention, combining oncologic safety with enhanced recovery and cosmetic benefits. Further research is needed to standardize protocols, evaluate long-term outcomes, and define its role within broader personalized cancer prevention strategies.

## 1. Introduction

Over the past two decades, the management of gynecologic cancers has been significantly influenced by the recognition of hereditary cancer syndromes and the role of preventive surgery. For women carrying mutations in high-risk genes such as BRCA1, BRCA2, or mismatch repair genes associated with Lynch syndrome, risk-reducing salpingo-oophorectomy (RRSO) and hysterectomy are established strategies endorsed by major guidelines [1,2,3].

Traditionally performed via open or laparoscopic approaches, these procedures have evolved to offer improved safety and reduced morbidity. However, despite these advances, visible abdominal scars, postoperative pain, and longer recovery remain concerns, especially for younger, asymptomatic patients seeking preventive interventions [4,5].

Vaginal natural orifice transluminal endoscopic surgery (vNOTES) has recently emerged as an innovative, scarless surgical alternative that merges the benefits of laparoscopy with the minimal invasiveness of vaginal surgery [6]. While initially adopted in benign gynecology, its use is expanding into oncologic prophylaxis [7].

This narrative review explores the evolving role of vNOTES in risk-reducing gynecologic cancer surgery, with a focus on its application in hereditary cancer syndromes. We aim to synthesize current evidence, technical considerations, and implications for clinical practice.

## 2. Materials and Methods

This is a narrative review aimed at synthesizing current evidence on the use of vNOTES in the context of risk-reducing gynecologic cancer surgery, particularly for women with BRCA mutations or Lynch syndrome.

### 2.1. Search Strategy and Sources

We performed a structured literature search using the PubMed and Web of Science databases to identify relevant studies published from January 2000 to April 2025. The following search terms were used in various combinations: “vNOTES”, “vaginal natural orifice transluminal endoscopic surgery”, “risk-reducing surgery”, “salpingo-oophorectomy”, “hysterectomy”, “BRCA”, “Lynch syndrome”, and “hereditary gynecologic cancer”. Additional studies were identified by reviewing the reference lists of included articles.

### 2.2. Inclusion and Exclusion Criteria

We included studies that reported on the use of vNOTES in gynecologic surgery with specific relevance to risk-reducing or oncologic contexts, such as procedures performed in BRCA mutation carriers or women with Lynch syndrome. Eligible articles provided clinical outcome data, including complication rates, operative time, hospital stay, estimated blood loss, or patient-reported outcomes. Only original research articles—such as case reports, cohort studies, and clinical trials—published in English and indexed in PubMed or Web of Science were considered. Studies were excluded if they focused exclusively on benign indications without relevance to cancer prevention, lacked outcome data, were published only as conference abstracts or technical videos, or were not peer-reviewed.

### 2.3. Study Selection

**Figure 1:**
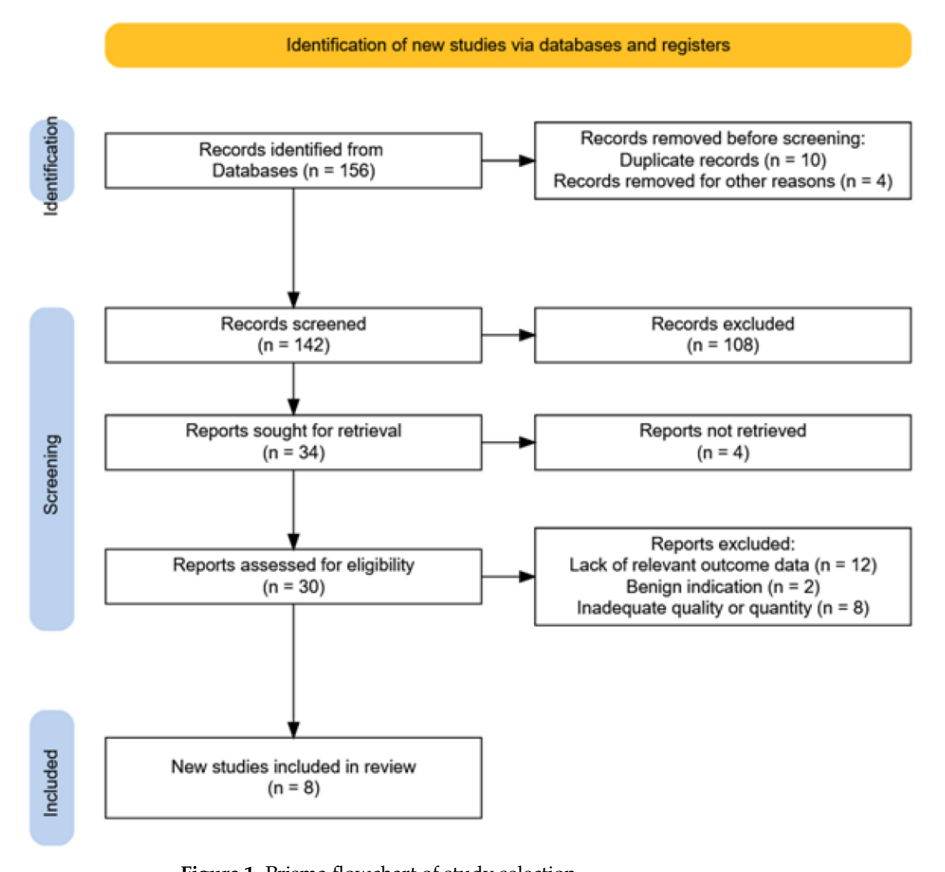
Prisma flowchart of study selection.

An initial total of 156 articles were identified. After removing duplicates and screening titles and abstracts, 30 full-text articles were assessed for eligibility. Of these, 16 articles met the preliminary inclusion criteria, and eight were ultimately included in the qualitative synthesis for their direct relevance to prophylactic gynecologic surgery using vNOTES (Figure 1).

Although this review follows a narrative design, a basic quality assessment of the included studies was performed to enhance transparency. We applied a simplified version of the ROBINS-I tool (Risk Of Bias In Non-randomized Studies of Interventions), assessing each included study based on the following domains: selection bias, outcome reporting, comparability of groups, and completeness of follow-up. Most included studies were retrospective and small in scale, often with limited follow-up and without control groups, which introduces a moderate-to-high risk of bias. The absence of randomization and heterogeneity in surgical expertise across centers were further limiting factors. Future systematic reviews should incorporate formal tools such as ROBIS or full ROBINS-I to better quantify study quality and evidence strength. Such limitations will further be elaborated.

### 2.4. Data Extraction and Synthesis

From each eligible study, we extracted information regarding the type of surgical procedure (e.g., hysterectomy, salpingo-oophorectomy), patient population (including mutation status), operative metrics (e.g., blood loss, operative time), complications, hospital stay, and postoperative recovery.

Where available, comparative outcomes with conventional laparoscopy or vaginal hysterectomy were highlighted. Particular attention was given to studies involving BRCA mutation carriers and women with Lynch syndrome undergoing risk-reducing procedures. Sectioning and Extensively Examining the FIMbriated End (SEE-FIM) protocol adherence was tracked when reported.

### 2.5. Review Design

Given the emerging nature of this surgical approach and the limited number of randomized trials, a narrative (non-systematic) review format was chosen to allow for thematic synthesis of clinical, surgical, and technical outcomes. Given the narrative nature of this review, a formal risk-of-bias assessment tool (such as the Cochrane Risk of Bias tool or ROBINS-I for non-randomized studies) was not applied. While care was taken to include high-quality and peer-reviewed literature, this limitation should be acknowledged, and future systematic reviews may benefit from structured quality appraisal frameworks to more rigorously evaluate evidence strength.

## 3. Overview of Hereditary Gynecologic Cancer Syndromes

Hereditary gynecologic cancers account for a small but significant subset of malignancies affecting women, and for those who carry genetic mutations, the lifetime risk is dramatically elevated [8]. The most well-characterized hereditary syndromes in this context are Hereditary Breast and Ovarian Cancer (HBOC) syndrome—typically involving *BRCA1* and *BRCA2* mutations—and Lynch syndrome, which arises from germline mutations in one of several mismatch repair (MMR) genes, including *MLH1*, *MSH2*, *MSH6*, and *PMS2* [9].

In women with BRCA1 or BRCA2 mutations, the lifetime risk of ovarian cancer ranges from approximately 10% to 50%, depending on the specific gene and family history [10,11,12]. Importantly, the origin of these so-called “ovarian” cancers is now understood to lie, in many cases, within the fimbrial end of the fallopian tubes. This insight has redefined the anatomic targets of prophylactic intervention, with bilateral salpingo-oophorectomy (BSO) being the standard recommendation, usually between the ages of 35 and 45, and earlier if childbearing is complete [13,14]. These procedures not only significantly reduce the risk of high-grade serous carcinoma, but also confer a measurable reduction in breast cancer risk, particularly in premenopausal women [14].

In contrast, Lynch syndrome confers a distinct pattern of cancer risk. Women with this condition face a 30–60% lifetime risk of endometrial cancer and a 10–20% risk of ovarian cancer, typically arising at a younger age than in sporadic cases [15]. Unlike BRCA, where breast surveillance often substitutes for prophylactic mastectomy, the risk of endometrial cancer in Lynch syndrome is best mitigated through surgical removal of the uterus and adnexa [16,17]. Guidelines from major societies such as NCCN and ESGO recommend considering prophylactic hysterectomy with BSO by the end of childbearing, especially if the patient is undergoing abdominal or pelvic surgery for another indication [18,19].

It is precisely within this delicate clinical picture that vNOTES has the potential to offer an ideal solution. As a scarless, endoscopic approach capable of meeting the oncologic objectives of prophylactic surgery, vNOTES may reduce the psychological and physical barriers that currently delay or deter risk-reducing interventions.

## 4. Principles and Technical Aspects of vNOTES

At its core, vNOTES provides direct access to the peritoneal cavity through the posterior vaginal fornix. The approach is both anatomically elegant and technically innovative, bypassing the need for abdominal incisions while preserving the visual and instrumental advantages of endoscopic surgery.

The procedure begins with a colpotomy, typically in the posterior fornix, through which a multi-channel single-port device is installed. Carbon dioxide insufflation creates pneumoperitoneum, and a laparoscopic camera provides visualization. Through the same port, standard laparoscopic instruments are used to perform adnexal surgery, hysterectomy, or combined procedures, with surgical steps nearly identical to those of transabdominal laparoscopy [20].

In risk-reducing surgery, vNOTES must be adapted to meet oncologic standards. A clear view of the pelvic and lower abdominal peritoneum is essential, especially in BRCA carriers, where detailed inspection of the fimbrial ends of the fallopian tubes is critical due to the risk of occult serous tubal intraepithelial carcinoma lesions (STICs) [13]. Regarding tissue processing, we emphasize the SEE-FIM, which is a protocol used to detect early, non-invasive precancerous lesions—particularly STICs—in the fallopian tubes of high-risk women, such as BRCA mutation carriers. It involves amputating the fimbrial (distal) end of the tube, serially sectioning it at 2–3 mm intervals, embedding all slices in paraffin, and performing detailed histologic examination. This method increases the likelihood of identifying microscopic lesions that may otherwise go undetected with routine sectioning [21].

Tissue retrieval must follow strict protocols; all specimens should be placed in endobags before removal to prevent potential cancer cell spillage [22,23].

**Figure 2:**
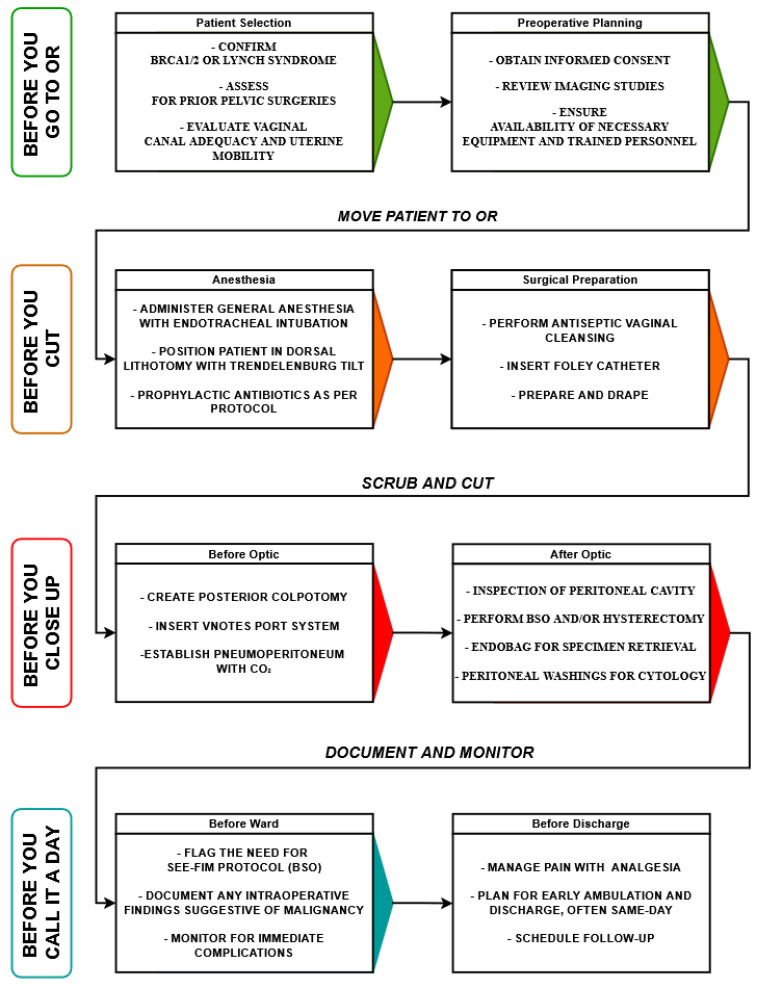
vNOTES procedural workflow for risk-reducing gynecologic surgery.

To ensure safe and standardized implementation of vNOTES in risk-reducing gynecologic surgery, a procedural workflow has been proposed and summarized in Figure 2.

The diagram follows a stepwise patient journey from preoperative assessment to postoperative discharge. Key stages are color-coded and grouped by surgical phases: Before You Go to OR includes patient selection and preoperative planning, ensuring appropriate genetic confirmation, surgical risk assessment, and logistical preparation. Before You Cut focuses on anesthesia induction, patient positioning, prophylactic measures, and surgical field preparation. Before You Close Up delineates intraoperative steps divided into pre-optic and post-optic phases, highlighting port insertion, peritoneal inspection, specimen retrieval in endobags, and cytological sampling. Before You Call it a Day emphasizes critical oncologic steps, including adherence to the SEE-FIM protocol and documentation of findings, along with postoperative care, pain management, early mobilization, discharge planning, and follow-up scheduling. This structured pathway reinforces procedural safety, oncologic rigor, and patient-centered recovery within vNOTES.

## 5. Clinical Applications of vNOTES in Prophylactic Oncology

### 5.1. RRSO in BRCA Mutation Carriers

RRSO reduces the risk of tubo-ovarian cancer by over 80% and modestly decreases breast cancer risk when performed before menopause. Several growing case series have demonstrated the feasibility and safety of vNOTES-RRSO in BRCA mutation carriers [1].

From the patient perspective, vNOTES offers significant benefits in terms of postoperative recovery and satisfaction. One prospective cohort study of BRCA carriers undergoing vNOTES-RRSO reported that over 90% of patients were discharged within 12 h postoperatively, with significantly lower analgesic requirements compared to matched laparoscopic controls [24]. Moreover, the cosmetic advantage of a truly scarless approach is highly valued by this younger patient population.

### 5.2. Prophylactic Hysterectomy ± BSO in Lynch Syndrome

While risk-reducing surgery in BRCA mutation carriers primarily targets the adnexa, women with Lynch syndrome face a distinct cancer risk profile, including a markedly increased lifetime risk of both endometrial and ovarian cancer. Consequently, prophylactic hysterectomy with or without BSO is recommended once childbearing is complete [25,26,27].

Traditionally performed via laparoscopic or robotic routes, hysterectomy has long been feasible via the vaginal approach. However, conventional vaginal hysterectomy can be limited by uterine size, prior surgeries, or lack of descent.

In the specific context of Lynch syndrome, where lymphadenectomy is not routinely indicated in purely preventive surgeries, vNOTES hysterectomy with or without BSO offers a safe and patient-centered alternative. Early feasibility studies suggest that vNOTES can achieve comparable operative times, complication rates, and patient outcomes to conventional laparoscopy [28,29].

## 6. Surgical Outcomes, Oncologic Safety, and Summary of Key Clinical Studies

As with any emerging surgical approach, the credibility of vNOTES in the setting of risk-reducing gynecologic oncology depends on its ability to match the oncologic safety and surgical efficacy established by conventional minimally invasive methods [1,30,31].

Below is a synthesis of selected studies that provide valuable outcome data—ranging from surgical safety to quality of life—and offer comparison points with traditional laparoscopic or vaginal approaches (Table 1).

In a large-scale, multicenter observational study, Housmans et al. (2020) reported outcomes from the International vNOTES Registry (iNOTES), which included over 1000 patients undergoing vNOTES procedures, the majority of which were hysterectomies. The overall complication rate was 5.2%, comprising 1.4% intraoperative and 3.8% postoperative complications. While blood loss and hospital stay were not consistently reported across all cases, the study confirmed the broad feasibility and acceptability of vNOTES in a wide patient population. Notably, the authors emphasized the low conversion rate and the reproducibility of the technique across multiple centers and surgeons [6].

Another prospective study conducted by Kim et al. (2024) compared 33 women undergoing vNOTES hysterectomy with 40 patients undergoing single-port access (SPA) laparoscopy, all performed by a single experienced surgeon. The groups were similar in age and BMI. The vNOTES group had significantly lower pain scores at 12 h postoperatively (mean VAS score: 2.7 vs. 4.2, *p* < 0.001). One ureteral injury occurred in the vNOTES group, while no intraoperative complications were reported in the SPA laparoscopy group. The findings support the conclusion that vNOTES may offer superior early postoperative pain control [32].

A meta-analysis of 13 studies including 1340 patients was published by Yang et al. (2019), directly comparing vNOTES to conventional laparoscopic surgery found that vNOTES was associated with significantly shorter operative times (mean difference: −10.2 min, *p* < 0.01), less blood loss (mean difference: −28.5 mL, *p* < 0.01), and shorter hospital stay (mean difference: −0.6 days, *p* = 0.03). Additionally, vNOTES patients experienced lower pain scores on postoperative days 1 through 3 (*p* < 0.001). Importantly, no significant differences in complication rates were found between the two approaches, suggesting that vNOTES offers a safer, less invasive alternative without compromising outcomes [33].

In a randomized controlled trial comparing 30 patients undergoing vNOTES hysterectomy and 30 undergoing conventional vaginal hysterectomy (CVH), Lowenstein et al. (2024) reported that blood loss was significantly lower in the vNOTES group (mean: 60 mL vs. 143 mL, *p* < 0.01). No complications occurred in the vNOTES group, while 10% of patients in the CVH group experienced intraoperative or early postoperative complications. While pain scores were similar between groups, the study found that vNOTES procedures had shorter operative durations and greater procedural precision, especially in patients requiring concurrent pelvic reconstruction [34].

Another retrospective study by Goldenberg et al. (2022) included 46 patients, some of whom were BRCA mutation carriers undergoing risk-reducing surgery. The overall complication rate was 4.3%, with no major adverse events or conversions to open surgery. Though hospital stay and blood loss were not consistently reported, the authors emphasized the applicability of vNOTES in oncologic risk contexts, including cases with endometriosis and prior surgeries. The study supports the technique’s extension into more complex or high-risk surgical indications [35].

While not focused on oncologic prevention, one study by Liu et al. (2019) is valuable for its detailed assessment of postoperative quality of life following vNOTES sacrocolpopexy in 26 patients. Using the Pelvic Floor Impact Questionnaire, patients reported significant improvements in urinary and pelvic symptoms following vNOTES [36].

Lathouras et al. (2019) presented a case of a 52-year-old BRCA1 mutation carrier who underwent prophylactic BSO via vNOTES. The procedure was completed without complications, with intact specimen retrieval and same-day discharge. The patient reported high satisfaction with cosmetic and recovery outcomes, highlighting the feasibility of vNOTES for preventive surgery in high-risk patients [37].

Hurni et al. (2022) described two cases where vNOTES was used for surgical staging in presumed early-stage ovarian cancer. The transvaginal approach allowed for peritoneal washings, pelvic inspection, and biopsy collection without conversion. Although para-aortic access was limited, both procedures were completed safely, suggesting vNOTES may be applicable in selected diagnostic and early oncologic contexts [38].

Based on the current literature, we highlight the qualitative comparison between vNOTES and other surgical approaches (Table 2).

vNOTES offers notable advantages in cosmetic outcomes and postoperative recovery, with the added benefit of reduced instrumentation costs. While comprehensive cost-effectiveness analyses of vNOTES are still emerging, preliminary studies suggest potential economic advantages over conventional laparoscopic approaches. vNOTES may reduce operative times, hospital stays, and postoperative complications, leading to decreased healthcare costs. Additionally, the technique eliminates the need for abdominal incisions, potentially reducing the use of disposable instruments and associated expenses. However, some analyses indicate that initial procedural costs for vNOTES can be higher than those for traditional laparoscopy, underscoring the need for further research to assess long-term cost-effectiveness [39].

However, it does have limitations in accessing upper abdominal structures, which may restrict its application in staging or advanced oncologic procedures. In contrast, laparoscopy and robotic-assisted surgery offer broad anatomic access but at higher cost and resource intensity. Conventional vaginal surgery remains cost-effective and cosmetically favorable but may be limited by anatomic constraints and reduced intraoperative visualization. These comparisons underscore the importance of tailoring surgical approaches based on patient risk profile, anatomical considerations, and institutional capabilities.

Another key challenge is the learning curve, which requires proficiency in both vaginal and laparoscopic surgery. While data suggest that proficiency can be reached after approximately 20–30 cases, access to formalized training programs remains limited and inconsistent across institutions [30,40,41].

While the current review focuses on the application of vNOTES in prophylactic gynecologic oncology, it is important to acknowledge ongoing debate regarding its broader use in diagnostic or therapeutic oncologic surgery. Technical limitations, such as restricted access to the retroperitoneum and upper abdominal quadrants, present significant challenges—particularly in the presence of adhesions, endometriosis, or peritoneal carcinomatosis. These anatomical constraints may compromise peritoneal inspection or lymph node assessment, thereby limiting the oncologic completeness of staging procedures. Until further data emerge, its use should remain restricted to well-selected patients and primarily for prophylactic purposes, where oncologic staging is not required.

## 7. Limitations and Future Prospects

Despite the promising evidence supporting the feasibility and safety of vNOTES in risk-reducing gynecologic surgery, several important limitations must be acknowledged. First, the current body of literature is predominantly composed of retrospective series, case reports, and small prospective cohorts, with a notable paucity of randomized controlled trials specifically evaluating vNOTES in hereditary cancer prevention contexts. This limits the generalizability and strength of the conclusions that can be drawn, particularly regarding long-term oncologic outcomes and recurrence rates. Furthermore, the absence of structured, long-term follow-up data regarding patient-centered outcomes such as sexual function, pelvic floor integrity, and quality of life after vNOTES in risk-reducing contexts remains a critical gap. These parameters are particularly relevant given the preventive, asymptomatic profile of the patient population undergoing such procedures. Looking forward, there is a clear need for well-designed, multicenter randomized trials comparing vNOTES with conventional laparoscopic or robotic approaches in risk-reducing surgery, with a focus on both oncologic and patient-reported outcomes. Additionally, integration of vNOTES into broader personalized cancer prevention strategies will require interdisciplinary collaboration, standardized procedural pathways, and health-economic evaluations to clarify its cost-effectiveness relative to established techniques.

A major limitation of this review is the small number of studies (*n* = 8) eligible for inclusion, which restricts the generalizability of our conclusions. Moreover, the included studies were heterogeneous in multiple domains: surgical approach (pure vNOTES vs. hybrid), patient populations (BRCA vs. Lynch vs. general oncologic), procedural type (RRSO, hysterectomy, or both), and follow-up durations. This heterogeneity precluded meaningful subgroup analyses or meta-analytical pooling. These limitations highlight the need for greater harmonization in future study designs and multicenter collaborations that enable more robust statistical comparisons, including stratified analyses by genetic mutation type, procedure type, and institutional surgical expertise.

## 8. Conclusions

vNOTES represents a promising advancement in minimally invasive approaches for risk-reducing gynecologic surgery in women with hereditary cancer syndromes. Current evidence supports its feasibility, safety, and favorable perioperative outcomes, including reduced postoperative pain, faster recovery, and high patient satisfaction, while maintaining adherence to oncologic surgical standards. Despite these advantages, limitations such as the learning curve and restricted upper abdominal access necessitate careful patient selection and, in some cases, hybrid approaches. Further research is warranted to standardize surgical protocols, assess long-term oncologic safety, and clarify the cost-effectiveness of vNOTES in this preventive context. As surgical strategies continue to evolve within personalized cancer prevention programs, vNOTES may offer an important, patient-centered option that aligns with both surgical and psychosocial priorities.

## Figures and Tables

**Table 1:** Summary of key studies evaluating vNOTES in gynecologic surgery.

No.	Study and Year	Surgical Approach	No. of Patients	Complication Rate	Hospital Stay	Estimated Blood Loss	Quality of Life/Pain
1	Housmans et al. (2020) [6]	vNOTES	1000	5.2% (1.4% intraop., 3.8% postop)	Not specified	Not specified	Not specified
2	Kim et al. (2024) [32]	vNOTES vs. SPA Laparoscopy	33 (vNOTES), 40 (SPA)	1 ureteral injury (vNOTES)	Not specified	Not specified	Lower pain in vNOTES
3	Yang et al. (2019) [33]	vNOTES vs. Laparoscopy	1340 (meta-analysis)	*p* > 0.05	Shorter with vNOTES	Lower in vNOTES	Lower pain in vNOTES
4	Lowenstein et al. (2024) [34]	vNOTES vs. CVH	30 (vNOTES), 30 (CVH)	0% (vNOTES) vs. 10% (CVH)	Not specified	60 mL (vNOTES) vs. 143 mL (CVH)	Similar between groups
5	Goldenberg et al. (2022) [35]	vNOTES	46	4.3%	Not specified	Not specified	Not specified
6	Liu et al. (2019) [36]	vNOTES Sacrocolpopexy	26	0%	Not specified	30.87 ± 20.8 mL	Improved QoL (PFIQ)
7	Lathouras et al. (2019) [37]	vNOTES	1 (case report)	0%	Not specified	Not specified	Not specified
8	Hurni et al. (2022) [38]	vNOTES	2 (case reports)	0%	Not specified	Not specified	Not specified

SPA—Single-Port Access; CVH—Conventional Vaginal Hysterectomy; QoL—Quality of Life; EBL—Estimated Blood Loss. Estimates are based on aggregated data from the included studies. Comparative values are descriptive and should be interpreted with the heterogeneity of study designs and patient populations in mind.

**Table 2:** Qualitative comparisons between vNOTES and other surgical approaches.

	vNOTES	Laparoscopy	Robotic Surgery	Conventional Vaginal Surgery
**Access Route**	Transvaginal (natural orifice)	Transabdominal (multi-port)	Transabdominal (robot-assisted)	Transvaginal (manual)
**Cosmetic Outcome**	No visible scars	Multiple small scars	Multiple small scars	No visible scars
**Postoperative Pain**	Lowest among all methods	Low to moderate	Low	Low
**Hospital Stay**	Often same-day discharge	1–2 days typical	1–2 days typical	Same-day or next-day
**Instrumentation Cost**	Low (no robotic console)	Moderate (laparoscopic towers, trocars)	High (robot, instruments, maintenance)	Very low (no lap tower required)
**Anatomic Limitations**	Limited upper abdominal access	Broad access to abdomen	Broad access to abdomen	Difficult in nulliparous or adhesions
**Training Availability**	Moderate (emerging in training centers)	Widespread	Limited to high-resource centers	Widely taught
**Oncologic Safety Potential**	Requires strict emerging protocols	High, well-established	High, data emerging in prophylaxis	Limited by visualization and control

SEE-FIM—Sectioning and Extensively Examining the FIMbriated End; RRSO—Risk-Reducing Salpingo-Oophorectomy. This checklist is adapted from surgical standards reported in the literature and expert consensus, aiming to standardize vNOTES in hereditary gynecologic cancer prevention.

## Data Availability

The raw data supporting the conclusions and results of this article will be made available on request.

## Abbreviations

vNOTES	Vaginal Natural Orifice Transluminal Endoscopic Surgery
BRCA	Breast Cancer gene (BRCA1, BRCA2)
RRSO	Risk-Reducing Salpingo-Oophorectomy
SEE-FIM	Sectioning and Extensively Examining the FIMbriated End
STIC	Serous Tubal Intraepithelial Carcinoma
BSO	Bilateral Salpingo-Oophorectomy
HBOC	Hereditary Breast and Ovarian Cancer
MMR	Mismatch Repair
NCCN	National Comprehensive Cancer Network
ESGO	European Society of Gynecological Oncology
SPA	Single-Port Access
CVH	Conventional Vaginal Hysterectomy
QoL	Quality of Life
VAS	Visual Analog Scale
PFIQ	Pelvic Floor Impact Questionnaire
RCT	Randomized Controlled Trial
iNOTES	International vNOTES Registry
